# Characterizing disease states from topological properties of transcriptional regulatory networks

**DOI:** 10.1186/1471-2105-7-236

**Published:** 2006-05-02

**Authors:** David P Tuck, Harriet M Kluger, Yuval Kluger

**Affiliations:** 1 Department of Pathology, Yale University School of Medicine, New Haven, Connecticut 06510, USA; 2 Department of Internat Medicine, Yale University School of Medicine, New Haven, Connecticut 06510, USA; 3 Department of Cell Biology, New York University School of Medicine, New York, New York 10016, USA

## Abstract

**Background:**

High throughput gene expression experiments yield large amounts of data that can augment our understanding of disease processes, in addition to classifying samples. Here we present new paradigms of data Separation based on construction of transcriptional regulatory networks for normal and abnormal cells using sequence predictions, literature based data and gene expression studies. We analyzed expression datasets from a number of diseased and normal cells, including different types of acute leukemia, and breast cancer with variable clinical outcome.

**Results:**

We constructed sample-specific regulatory networks to identify links between transcription factors (TFs) and regulated genes that differentiate between healthy and diseased states. This approach carries the advantage of identifying key transcription factor-gene pairs with differential activity between healthy and diseased states rather than merely using gene expression profiles, thus alluding to processes that may be involved in gene deregulation. We then generalized this approach by studying simultaneous changes in functionality of multiple regulatory links pointing to a regulated gene or emanating from one TF (or changes in gene centrality defined by its in-degree or out-degree measures, respectively). We found that samples can often be separated based on these measures of gene centrality more robustly than using individual links.

We examined distributions of distances (the number of links needed to traverse the path between each pair of genes) in the transcriptional networks for gene subsets whose collective expression profiles could best separate each dataset into predefined groups. We found that genes that optimally classify samples are concentrated in neighborhoods in the gene regulatory networks. This suggests that genes that are deregulated in diseased states exhibit a remarkable degree of connectivity.

**Conclusion:**

Transcription factor-regulated gene links and centrality of genes on transcriptional networks can be used to differentiate between cell types. Transcriptional network blueprints can be used as a basis for further research into gene deregulation in diseased states.

## Background

The study of mammalian transcription based on high throughput gene expression data has primarily focused on the identification of individual differentially expressed genes, co-regulated gene sets and genes with inferred functional similarity based on co-expression under various conditions. Investigators have identified functional modules from gene expression data using a reverse-engineering approach to reveal regulatory subunits, based on probabilistic graphical models [1], singular value decomposition [2-4] and network component analysis [5], as well as various other methods. Methodologies for reconstructing and inferring elements of genetic and metabolic networks [1,6-11], identifying new gene modules [12-14] and statistically characterizing topological network features are the focus of much research [15-19], especially for model organisms. Recent advances in network analysis have focused on topological changes and static and dynamical network properties in yeast and E coli [20,21].

There has been extensive study of expression patterns of genes which can discriminate between normal and cancer specimens, separate different tumor types or predict clinical outcome (for example [22-24]), including in the context of networks [25,26]. The study of transcriptional networks has been facilitated by the incorporation of both DNA-protein binding data [27-31] as well as the computational prediction of binding sites, based primarily on methods using position weighted matrices (PWM) [32-34].

In the present work we constructed human transcriptional regulatory networks of the whole genome by combining evidence of co-expression in microarray datasets with transcription factor-gene regulatory relationships based on sequence predictions and literature based evidence. In these studies we revealed condition specific (phenotype) networks in order to discover network features that can shed light on unique transcriptional processes associated with a particular phenotype. We studied the extent to which networks vary between different phenotypes. Specifically, we explored network features in different cell types such as normal versus cancer specimens, and in specimens from patients whose disease recurs versus those remaining disease free.

## Results

### Network construction

**Figure 1:**
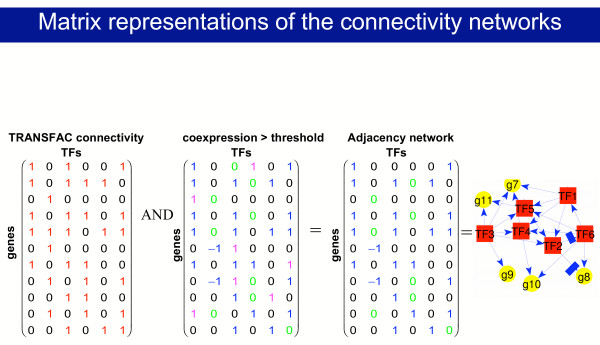
Matrix representation of the reconstructed regulatory network. A non-zero value at the intersection between a TF column and a gene row in the connectivity matrix represents a literature-mined (stored in TRANSFAC) or putative binding of this TF to the 1 kb upstream region of the gene promoter (based on matching the TRANSFAC PWM with this promoter region). A value of +1 in the co-expression matrix derived from microarray data was assigned if the TF is up-regulated while the target gene is up-regulated, -1 if the TF is up-regulated while the target gene is down-regulated and 0 otherwise. When overlaying the connectivity and co-expression matrices (using element by element multiplication of these matrices as denoted by the "AND" operation above) we obtain the putative regulatory network represented by the adjacency matrix, whose blue nonzero entries represent both binding and co-expression between a TF and a gene, and whose green and black zero entries correspond to TFs and genes that are not bound and/or not co-expressed. On the right we show graphical representation of the adjacency network. The flow is depicted in the supplementary data [see Additional file 1].

We constructed regulatory networks by intersecting a connectivity network (representing TF binding to gene promoter regions) with co-expression networks (representing TF-target gene co-expression). Construction of these networks is depicted in Fig. 1. The connectivity network was derived by a) matching known TF binding sites to the promoter regions of genes (TRANSFAC [34,35]), and b) literature-based known TF-target gene interactions which are included in the TRANSFAC database. In addition, we note that limited ChlP-on-chip data have recently been included in TRANSFAC. For data derived from each microarray (for an individual sample or patient), we constructed a co-expression network, such that each TF-gene pair was assigned a co-expression value; -1 if the TF is up-regulated while the target gene is down-regulated, +1 if they are both up-regulated and 0 otherwise. Condition specific (CS) transcriptional regulatory networks are derived from the intersection between the connectivity and individual co-expression networks. Details of construction of the networks are described in the methods section below.

The CS networks we studied were drawn from six gene expression studies using three types of datasets; data derived from normal cell lineages [36], tumors versus normal tissues [37], and disease-specific tumors associated with variable clinical outcomes [38-41]. The sizes of the networks were dependent on the number of known genes in the particular microarray platform, and ranged from 4821 genes and 196 TFs on the earliest Affymetrix array to 13363 genes and 233 TFs on the Affymetrix U133A and B arrays. Different microarray platforms contain different sets of genes. To construct each of these CS networks, we used a subset (or sub-matrix) of the connectivity network that only contains the genes and TFs that are present in a particular microarray platform. The total "density" of these connectivity sub-networks (i.e. the fraction of putative TF-gene connections out of all possible connections for a particular sub-network) was very similar for all platforms, ranging between 4.6% and 5.1%. With the expression thresholds we chose for the CS network construction, the fraction of connections ranged from 0.3% to 1% in the CS networks.

We examined the robustness of our general results by studying a range of values for the parameters used in constructing the connectivity networks, including the extent of upstream promoter region sequences (between 1000 bp and 5000 bp upstream of the transcription start site), different TF binding profiles (the sets of position-weight matrices used to predict binding) as well as TRANSFAC thresholds for determining a protein-DNA binding site (see details in the Parameter Selection section below). All results reported here were generated by using sequences 1000 bp upstream of RefSeq identified transcription start sites and using a standard profile provided by TRANSFAC intended to minimize false positives.

### Classification based on regulatory network features

We hypothesized that each individual sample from a cohort of heterogeneous cancer patients has a distinct regulatory network, i.e., a pattern of activated links that gives rise to its gene expression profile. Based on this hypothesis, we explored how different aspects of network structure characterize different phenotypes.

#### Link-based classification

We introduced a network based classification approach that examines differences between patient samples by analyzing the activity status of regulatory links between genes. We constructed networks unique to individual patients, e.g. for individual patients with disease recurrence and for patients with no recurrence. This enables us to identify specific parts of the networks, which are differentially regulated between these conditions. The added value of this network-based classification is our ability to identify coordinated co-regulation of TF-gene pairs that are present only in survivors or only in non-survivors.

In the link-based approach, we attempted to discriminate between samples using differential TF-gene activity instead of using standard discrimination by gene expression profiling. A network is comprised of links and nodes. In our link-based approach, the activities of a network's links, which represent a TF regulating a gene, are used for classification, instead of using properties of the individual nodes.

#### Degree-based classification

We further classified our samples by using another topological property of the networks, the "centrality" of individual genes in the networks. Several measures of centrality have been defined [42]; here we report results using a centrality measure defined by the degree of nodes in the regulatory networks, the number of TFs activating or suppressing a particular gene (in degree) or number of genes regulated by a single TF (out degree). Specifically, instead of characterizing a sample by a gene expression profile, we use a sample's genome wide degree profile. We surmise that identifying the nodes with the largest changes in gene centrality (rewiring) will assist us in detecting hotspots associated with deregulation, leading to understanding disease processes at a transcriptional level.

#### Sample classification

For each of the datasets described we created regulatory networks for every sample and applied a meta-classifier (see Methods) that in the first step ranks the features (using an information gain measure) to identify the set of TF-gene links that collectively separate between classes (e.g., acute myeloid and acute lymphocytic leukemia populations). Our purpose was to find network features that classify different phenotypes, rather than to derive a classifier that is superior to commonly used classifiers based solely on gene profiling. We reasoned that the discriminating network features would be useful to explore changes in regulatory mechanisms. Nevertheless, our link-based classifiers compared favorably with gene profiling classifiers.

Using the training sets, we performed feature selection to rank the links, genes and degree of the nodes that undergo the most substantial change between two phenotypes such as distinct blood cell types, different categories of leukemia, normal kidney versus renal carcinoma and breast cancer patients whose disease recurs versus those remaining disease free. Specifically, we studied three groups of datasets. In the first group we studied the following two-class datasets: a) acute lymphoblastic leukemia versus acute myeloid leukemia [23], b) two different myeloid leukemia types defined by the presence of two oncogeneic fusion proteins (AML1-ETO, t(8;21) versus RAR-PML, t(15;17)) [43]. In the second group, we analyzed several breast cancer datasets involving heterogeneous populations of patients with different outcomes (patients who had disease recurrence versus those that did not) [38,40,41,44]. The third group includes different matched cell types (normal kidney versus renal cell carcinoma each from the same patients [37], and normal monocytes versus normal polymorphonuclear leukocytes [36]).

To perform the link-based classification, we then passed the top links (features that were found to have the highest rank defined by the highest information gain in explaining the class label of the training set) to train a base classifier (nearest neighbors, decision tree, Naïve Bayes, etc.) using the training data only. To estimate the cross validation error rate of the classifier in the reduced feature space, we performed ten-fold cross validation ten times using ten different seeds. We note that for error estimation, cross-validation is performed with features selected from the training set only at each step in the cross-validation procedure, to avoid an optimistic bias in the accuracy estimates [45]. To compare the performance of the link-based classification an expression based analysis for identification of differentially expressed genes has been implemented using equivalent feature selection and machine learning procedures. Similarly, we formed a genome-wide profile indicating the in-degree (centrality) for all genes or out-degree for all TFs in each individual network, and searched for the subset of genes or TFs whose centrality measure is altered significantly between the two classes. The results using the same procedures for these three types of input data (link profiles, gene profiles and gene centrality profiles) are presented in Tables 1, 2.

For the leukemia datasets, linked-based sample separation of different lineages (ALL versus AML), and of two types of myeloid leukemia, was quite effective, with estimated classification error rates of 9% and 6%, respectively. Likewise, we were able to partition these data based on the genome-wide in degree profile of all the nodes in the regulatory networks with cross validated classification errors of 4% and 0% respectively, similar to error rate estimates obtained using gene expression levels of individual genes.

For the breast cancer datasets, the classification performance based on links, expression profiles or gene centralities was much weaker with error rates ranging from 17%-25% in the Duke dataset to 39%-43% in the Dutch dataset, reflecting the increased tumor heterogeneity in these populations. Due to the fact that many links in the connectivity network are inferred by using *in silico *matching between PWMs and promoter regions and the additional discretization (cutoff) steps involved in the construction of the CS regulatory networks, we may add some noise to the data, and expect some information loss respectively. Indeed, in some datasets the observed error rates obtained when using the link-based classifiers are slightly higher than those obtained by gene profiling, but are still in the same ballpark. However, when we classified the datasets using the in-degree profiles (and occasionally the out-degree profiles) we observed that in some datasets the error rates are slightly lower than those obtained by gene profiling. The degree of a gene is defined by the number of links associated with it, and therefore the collective information about the condition specific activity of multiple links associated with a gene may compensate the potential sources of noise and loss of information mentioned above.

For the datasets composed of distinctly different cell types, the network-wide status of link activities enabled us to cleanly discriminate between these cells with 0% cross validation error rates. Equivalent error rates with ten fold cross-validation were also obtained using the CS gene degree profiles or gene expression profiles.

**Figure 2:**
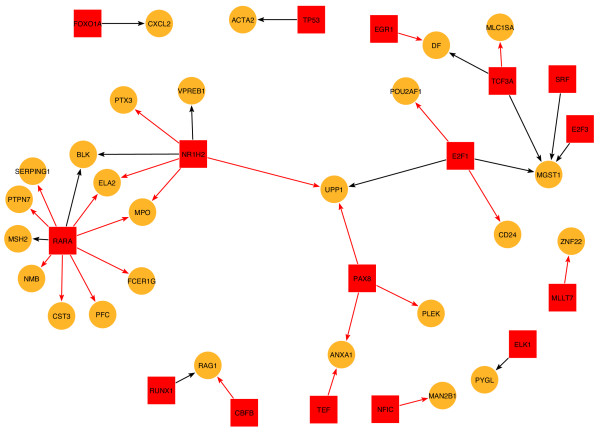
A combined set of links with the highest change in functional activity between patients with AML versus patients with ALL. To identify differential transcriptional activities between networks obtained from two sample populations, we analyzed the data matrix whose columns represent the individual adjacency networks and whose rows represent TF-gene link activities across the samples. Entries of this matrix can take one of three values; activation (1), inhibition (-1), no effect or undetermined relationship (0). The graph shown summarizes the output from a feature selection procedure (see methods section) for identifying the TF-gene regulatory links that have the most prominent differential transcriptional activities between AML and ALL patients. Target genes are denoted by yellow circles and TFs by red squares. Stimulatory and inhibitory regulatory links, are indicated by red and blue arrows respectively.

We display the differences between the regulatory network structures by drawing a graph which includes the top differentially activated links that discriminated between the two classes, e.g. the AML and ALL. We note that the network structures in each of these states contain many more regulatory links (many of them are common to both states), which makes them inscrutable upon visual display, whereas a graph as the one shown in Fig. 2 highlights the links that undergo changes between the networks that correspond to the two phenotypes.

For example, this graph suggests that the regulation of RAG1 (recombination activating gene 1) by RUNX1/CBFB (which comprises the subunits of the heterodimeric transcription factor, core binding factor) is a crucial link differentiating AML and ALL. RAG1 is known to be expressed in a stage-specific manner in various types of ALL (both T and pre-B) [46]. Yannoutsos et al recently demonstrated that an intergenic silencer which suppresses expression of RAG1 in developing lymphocytes is dependent on occupation of the RUNX binding sites [47]. The regulation by RARA (a member of the retinoic acid receptor family of transcription factors) of a set of genes involved in myeloid maturation is also identified (Fig. 2). RARA is known to be up-regulated in some types of AML [48].

Using a separate leukemia dataset we examined the differences in regulatory network structures which best discriminated a set of leukemia samples identified by translocations involving the AML1-ETO fusion gene versus those with translocations involving the PML-RAR fusion gene. Key links, which differentiated these two types of leukemia, included the regulation of the fusion protein RUNX1T1. We note that our algorithm would not be able to distinguish whether abnormal regulation of a gene in one class was due to mutations or epigenetic modifications of the target gene, as opposed to a change in cis-regulatory transcriptional control.

**Figure 3:**
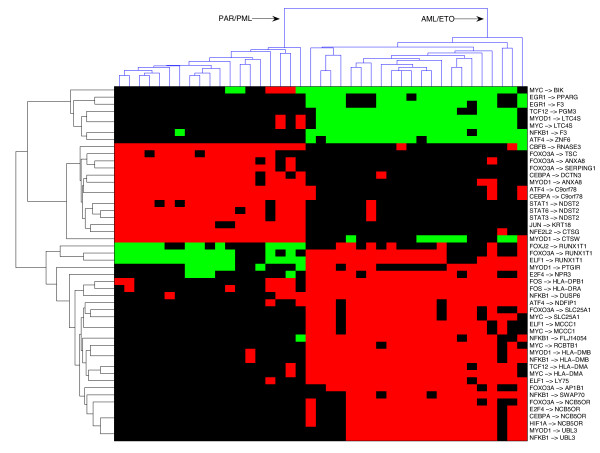
Classification utilizing differences in condition-specific network topologies (links). The links presented on the y-axis were extracted using a supervised learning scheme that identifies the TF-gene links that undergo the most substantial regulatory alterations between two classes of AML patients; those with a translocation resulting in the AML/ETO fusion protein versus those with the RAR/PML fusion protein. The red, black and green represent a stimulatory relationship, no relationship or inhibitory relationship of the TF-gene links, respectively.

**Figure 4:**
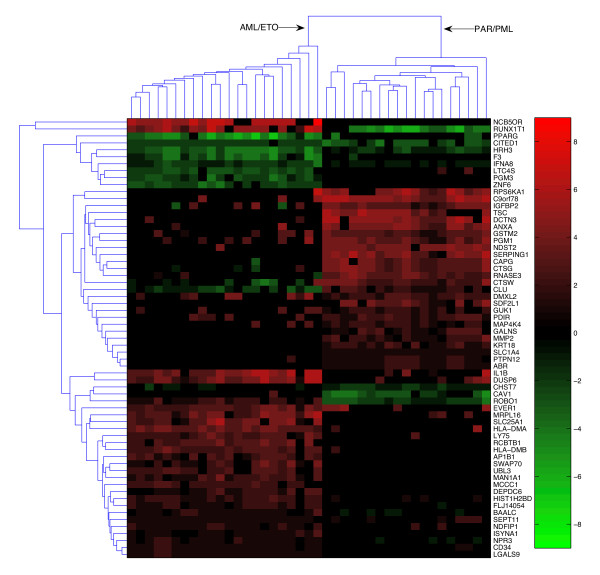
Classification utilizing differences in condition-specific network topologies (in-degree). The genes presented on the y-axis were extracted as in Fig. 4 using a similar machine learning procedure that identifies genes whose in-degree (the number of regulators that control these genes) undergoes the largest change or rewiring.

In analogy to standard microarray analyses in which the genes that separate two populations are presented in heatmaps, we identify and display TF-gene pairs that are predominantly linked in networks associated with one of the classes, but not in the networks of the other class. Table 2 shows examples of these differentially active links. Fig. 3 shows the separation of AML samples using the combined TF-gene pair activities. For example the regulatory TF-gene pair Myc→HLA-DMA is more active in AML involving the fusion protein AML/ETO than in AML involving the fusion protein RAR/PML. The binding of Myc to HLA-DMA has been demonstrated in a study of the regulatory role for c-Myc in Burkitt's lymphoma cells [29]. Fig. 4 shows the separation of the same AML populations using a classification scheme based on the changes (rewiring) of the in-degree of all genes. We note that the in/out degree measure of a given node is obtained by summing the relevant row/column of the regulatory network, and therefore it can be positive or negative. This is determined by whether the regulators that control the target gene (node) are mostly stimulatory or inhibitory.

Our analysis suggests that a direct link from NFKB1 to F3 (coagulation factor III/Tissue factor) is among the top links that discriminate between the two types of myeloid leukemia. Constitutive expression of F3 by acute promyelocytic leukemia (APL) cells is thought to contribute to the common coagulation complications of this disorder. The expression of the APL specific PML/RARalpha fusion oncoprotein results in induction of F3 mRNA and promoter activity. Djordjevic et al [49] have recently drawn attention to the role of NFKB binding sites in the promoter region of F3, using human smooth muscle cells. They demonstrated that F3 mRNA and protein expression and surface procoagulant activity were increased in response to thrombin, primarily involving a sequence between -636 and -111 bp containing a distal, nuclear factor-kappaB (NFkappaB)-dependent element. These findings raise the possibility that this direct link of NFKB→F3 could also be highly relevant to the F3 mRNA and protein expression and procoagulant activity involved in the clinically important coagulopathy in APL. The link between EGR1→F3 also was noted to be important in our analysis. The regulation of TG by EGR-1 has been well documented [50], although its relevance in leukemia has yet to be determined.

In Fig. 3 we show only links with an information gain score > 0.5. Another regulatory link identified as an important discriminator between the two types of leukemia, with a score just below this cutoff, involves the C/EBP alpha (CCAAT/enhancer binding protein, CEBPA) regulating PPAR-gamma (PPARG). CEBPA is a transcription factor known to be involved in regulating granulocytic differentiation and proliferation of myeloid progenitors (reviewed in [51]). It is downregulated by the AML1-ETO fusion transcript in t(8,21) leukemia. Furthermore, its role in regulating PPARG (peroxisome proliferative activated receptor, gamma) is well documented in adipocyte development and bone marrow stromal cells. Although the relationship is highly complex and involves other C/EBP factors, there is strong evidence of direct regulation of PPARG [52]. Our findings suggest that the role of this direct link may deserve further evaluation in leukemia.

#### Parameter selection

We examined the robustness of our results by studying a range of values for the parameters used in constructing the connectivity networks and performing classification based on network features.

The parameters we used to determine up- or down-regulation in the co-expression networks in this work were at the 80^th ^and 20^th ^percentile of expression levels respectively or, for Affymetrix data, at absolute expression intensities of 200 and 50. To demonstrate the effect of these cutoffs on the classification performance we studied the leukemia dataset. Table 3 shows how the classification error rates depend on these parameters. In cases in which we use a ranking procedure to select the most discriminative features (link, centralities), we show that networks generated using more extreme cutoffs (e.g. 90^th ^and 10^th ^percentile) are associated with larger error rates. Networks generated using less extreme values than the 80^th ^and 20^th ^percentile (e.g. 66^th ^and 33^rd ^percentile) have similar error rates. We opted to use the 80^th ^and 20^th ^percentile cutoffs, as the frequency of false positive links in these less dense networks is lower. For the purposes of classification and localization analyses described below, we also studied CS networks using continuous co-expression and binding data. Nominal and continuous variables yielded similar results, as shown in Table 3. Our continuous co-expression matrix was constructed by preprocessing the gene expression data *g *by the following transformation: G = tanh[(g-μ)/δ], where μ is the average of the expression level of all genes across all experiments and δ is the corresponding inter-quartile range.

In Table 4 we show how variations in the parameters for connectivity network construction affect classification performance. The genomic region searched for TF binding sites was either 1000 bp or 5000 bp upstream of known genes. Two different collections of PWMs were used: 1) all the matrices provided by TRANSFAC relevant to mammalian genes, or 2) the selection of PWMs identified by TRANSFAC as 'high quality'.

### Cross-platform analysis

We examined whether the differentiating features found in networks constructed using one microarray study also differentiate between the networks constructed using another independent microarray study performed on another microarray platform [53]. We studied two acute myeloid leukemia datasets [43,54]. Although one of the datasets was small (9 cases), they both contained samples from untreated patients at initial diagnosis with well-defined subtypes based on the presence of well-documented fusion genes. In addition they used closely related platforms (Affymetrix U95 and U133A), for which most of the genes represented in the probe-sets for the older generation array are also represented in the newer U133A chip that was used in the larger dataset. We trained a classifier on the larger dataset by constructing networks using the subset of genes that was present on both the U95 and U133A platforms and used the top separating features to test its performance on the smaller leukemia dataset using U95 microarray gene profiles. We found that the cross- platform error rate was 11%, which is only 2% more than within platform cross validation. We note that many probesets, which allowed perfect separation of subtypes on the larger dataset, are missing on the older array, and therefore the performance of the classifier based on the subset of U95 genes is a bit inferior to the classifier that utilizes all the genes on the U133 chip. Interestingly, the network features actually performed somewhat better than gene expression profiles alone in the cross-platform evaluation. Although the sample size is small and we do not claim that network features are superior to gene expression levels, this adds additional support for the conclusion that there is useful information in the network features.

### Network proximity of gene sets

**Figure 5:**
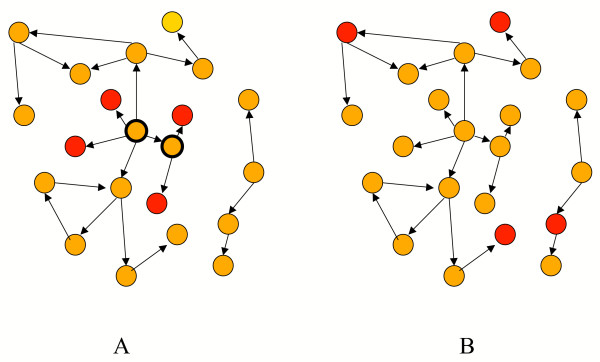
A scheme of two potential scenarios of regulatory networks. Genes that separate two classes of patients are denoted by red circles and TFs by yellow circles. A) The four separating genes are localized in the network such that the (link) distance between each pair is at most three. These four genes share two regulators denoted by black bold circumferences. These two regulators are likely to be relevant for sample discrimination because they regulate the four most discriminatory genes. B) The four separating genes have remote or no regulatory linkage and therefore their regulating TFs are less likely to be helpful in sample separation.

We subsequently explored whether differentially expressed genes are close neighbors in these transcriptional regulatory networks. We studied the dispersion or localization of differentially expressed gene sets in the network, as illustrated in the schema of Fig. 5. Our rationale was that concentration of these genes in focal regions, rather than delocalization over the entire network, might suggest strategies for interventions or further experimentation, not apparent from a perspective outside of the network context. Although prior functional relationships among genes may not be annotated in existing databases, they may share common regulators that affect them directly or indirectly via short regulatory paths within the condition specific (CS) network. We note that for the CS networks we used a binary version of the adjacency matrices, which only takes into account the presence or absence of a regulatory relationship between a TF and its target gene. To measure distances we did not differentiate between stimulatory and inhibitory relationships.

**Figure 6:**
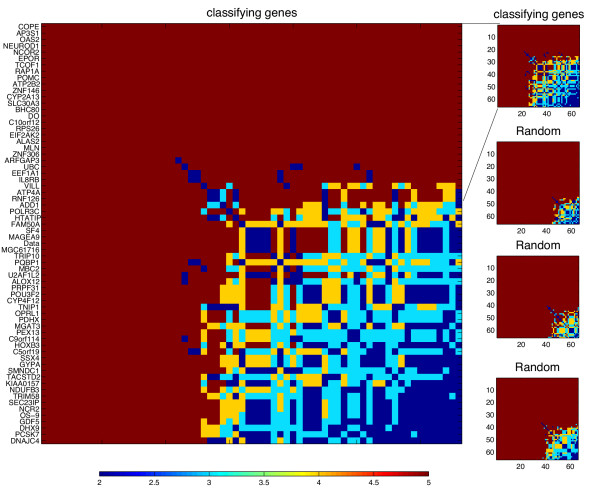
Distance map between the differentially expressed genes in a condition specific (CS) regulatory network. In this map we display the gene-gene distances between the pairs of genes that belong to the subset of differentially expressed genes. To determine the distance between gene *a *and gene *b*, we count the number of directed links leading from each TF to genes *a *and *b*, and calculate the sum of links of the two paths for each TF. The distance between genes is defined by the minimum of these sums (This also defines the common closest TF ancestor of this pair of genes). We show the distances between the pairs of genes that belong to the subset of differentially expressed genes separating between disease free, node-positive breast cancer patients and patients with recurrence [38]. This map resembles maps used in atlases to represent distances between different cities. The order of the genes on the x and y-axes is the same. The distances between them are color coded in the horizontal bar below the map (distances of 1 or 2 are indicated in blue, distance of 3 in cyan, distance of 4 in yellow and distances greater than 4 in Red. Distances > 4 typically represent disconnection, and in rare cases, long geodesic distances. In this example we identified 66 differentially expressed genes (see list on the y-axis) and found that a large subset of these genes are close to each other on the class collective CS network derived from the patients that have recurrence. This proximity is significant when compared to the proximity obtained by choosing a hundred same size random sets of genes (p < 0.01). This suggests a regulatory association between the differentially expressed genes. To demonstrate the tendency of these genes to be localized on the class collective CS network, we show three examples of distance maps of randomly selected, same size, sets of genes (lower three maps in the right column). The top map on the right of this figure is a scaled down map of the classifying genes, and is identical to the larger map on the left. This phenomenon of localization is not observed in the corresponding disease free collective CS network.

We first constructed a "class collective" CS network for each phenotype by aggregating individual networks derived from samples of the same type and retaining links that appear to be active in at least 25% of the samples. For instance, for the dataset of ALL and AML patients we derived two class collective CS networks, one representing the ALL patients and the other the AML patients. Subsequently, we extracted a subset of genes whose collective expression patterns differentiate between the classes (e.g., normal versus malignant renal cell, AML versus ALL, or poor outcome versus good outcome in breast cancer) for each microarray dataset. We then computed the geodesic distances between each pair of genes in the subset of differentially expressed genes (the minimal number of links needed to traverse the path between each pair of genes via a common set of regulators) in the class collective CS networks, e.g., in the ALL CS network and in the AML CS network. To examine whether the subset of differentially expressed genes share common regulators in one or both of the class collective CS networks we compared their distance distribution to the distance distributions of many random, same-size subsets of genes in these networks. Interestingly, the subset of differentially expressed genes tended to localize closely on the AML network (p < 0.01), when compared to the gene-gene distances of random sets of genes on the same class specific network. When constructing class collective networks for more heterogeneous datasets such as breast cancer patients with or without disease recurrence using the Duke dataset, we also found that the many of the differentially expressed genes were concentrated in neighborhoods on the network (p < 0.01) (Fig. 6).

**Figure 7:**
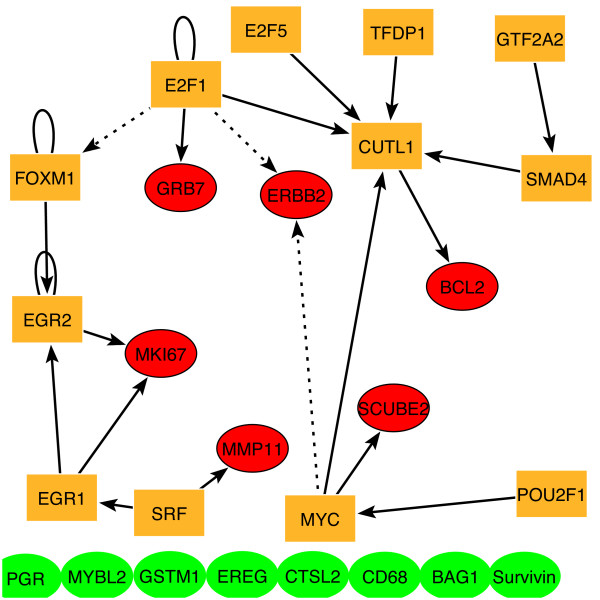
Breast cancer condition specific (CS) regulatory sub-network. We studied the Dutch datasets [39, 44, 55] to identify regulatory connections between sixteen genes that were recently implicated in recurrence in node-negative breast cancer [56]. To visualize regulatory distances between these genes and to identify the regulators involved in their co-alteration in the CS recurrence regulatory network, we extracted from this CS network a sub-network involving these genes and all the TFs that regulate them directly or indirectly. Fourteen of these genes are included in the Dutch datasets (red and green ovals). Six of the genes (red) form a highly connected and localized CS sub-network. TFs linked to any of these genes directly or indirectly are denoted by yellow boxes. Solid arrows stemming from Myc and E2F1 represent links supported by ChlP-on-chip experiments [29], whereas predicted links that were not matched by these non-breast cancer experimental data are shown with dotted arrows.

Similar results were obtained for the 295 breast cancer samples from the Dutch dataset [40,44,55] and for 286 breast cancer samples analyzed recently [41], where genes are more tightly localized in the CS network constructed from patients with recurrence (data not shown). In a recent study employing RT-PCR technology, a set of 16 genes was found to be valuable in predicting recurrence in node-negative, estrogen receptor positive, Tamoxifen-treated breast cancer patients [56]. Since the samples in the Dutch study also consisted of node-negative patients with breast cancer, it is reasonable to examine the regulatory distance distribution of this independent set of genes in the class collective network constructed from the Dutch dataset for patients with recurrence, as well as the network constructed from patients that were disease free. We found that genes from this set, including Bcl-2, ERBB2, GRB-7, Ki-67 and SCUBE-2, tend to localize on the Dutch class collective recurrence network (Fig. 7 (p < 0.01), but not on the disease free network. The close proximity of these genes in the recurrence network reveals "hot spots" of deregulation in cancer, not found in the non-recurrence network, and may suggest sub-networks which could be suitable targets for intervention or for further focused study.

One of the limitations in our definition of connectivity is that putative links between TFs and regulated genes are determined primarily by curated literature references or sequence similarity searches for potential DNA binding sites. The binding connectivity network does not include, for instance, high throughput binding data, which to date remain relatively limited for human cells. We explored the extent to which experimental validation of binding sites might alter our results by replacing connectivity data for the breast cancer subsets with ChlP-on-chip data for three TFs (Myc and E2F1), for which ChlP-on-chip data were available [29]. Although these studies were not performed using breast cancer samples, several predicted links between genes and regulators were substantiated by these experimental data (Fig. 7, solid arrows stemming from Myc and E2F1). The few predicted links that were not matched by these non-breast cancer experimental data are shown with dotted arrows in Fig. 7. Use of Chip-on-chip data from Burkitt's lymphoma experiments leads to a noticeable rewiring of the network, although the list of TFs that link between this set of 16 genes did not change when including the experimental ChlP-on-chip data. This rewiring could result from a) false positive predictions obtained by the MATCH program, b) the promoter regions printed on the chip do not completely overlap with the RefSeqs representing the gene, and therefore binding was not observed experimentally in the ChlP-on-chip studies, c) the binding targets of the factors investigated in the Burkitt's lymphoma cells might be somewhat different from the targets of these factors in the breast cancer samples.

## Discussion

There is an extensive body of literature proposing methods for classification of phenotypes based on genome-wide gene expression datasets, and many of these have potential for addressing clinical or biological questions in cancer research. In the present study we explored a novel alternative classification paradigm, using regulatory networks. The intersection of gene profiling analysis with an experimentally and computationally derived connectivity network of binding between TFs and their gene targets offers a pragmatic approach that enables us to get beyond identification of differentially expressed genes and decipher the alterations in transcriptional control associated with these genes.

Distinct cell types are typically separated by the collective expression profiles of genes identified using supervised learning. To gain new biological insights in microarray analysis several groups have proposed algorithms for integration of pathway databases with gene expression profiles [36,57-59]. In our earlier work [36] we concluded that differences between the distinct cell types are so pervasive that it is hard to implicate a small number of characteristic biological processes. In fact we attempted to classify the samples by using the gene expression profiles of one pathway at a time (i.e. by taking into account only the expression of genes that belong to this pathway) and observed that numerous pathways have differential expression behavior between the cell types. However, cohorts of heterogeneous cancer patients are much more difficult to separate than distinct cell types, even if one uses a subset of genes selected using supervised learning in a manner that attempts to minimize the classification error. In cohorts of cancer patients with different clinical outcome, pathway based classification leads to poorer performance (data not shown) than using standard supervised learning based on gene expression, and does not provide clear insights into disease dysregulation. Instead of combining pathway data with gene expression profiles, here we have attempted to integrate DNA binding databases, with a similar goal of gaining biological insight by inferring the specific conditions of activity of regulatory links and rewiring of transcriptional control of each node in the regulatory network.

In the current study, we hypothesized that each individual sample from a cohort of cancer patients has a distinct regulatory network, i.e., set of activated links, and that this regulatory network structure can be used for separating conditions. It is expected that network structures of patients from different phenotypes will differ more substantially than network structure within the same phenotype. The differences between these networks are reflected in their topological properties, e.g., the centrality of each gene in the network, the activity status of each link in the network, the Hamming distance between networks, to name just a few.

Other papers have demonstrated that network properties can vary between cancer and normal cells [25,26]. These networks were based on protein-protein interactions [26] or gene expression only [25], whereas in the current study we construct directed regulatory transcriptional networks with information on suppression versus stimulation. Here we constructed condition-specific networks for each individual patient, which we use for classification of patients within a disease population.

Classification based on topological changes in the networks allows us to implicate links that seem to be more active in one population than in the other populations. These putative links allow us to infer causal relationships between the TFs and their potential target genes, and therefore can provide us with hypotheses that are not apparent in straightforward or pathway assisted analysis of microarray experiments. Furthermore, by comparing the centrality of each gene across the regulatory networks of all patients pooled from two sub-populations (e.g. recurrence vs. disease free breast cancer patients), we can focus our attention on genes whose regulatory control (or their rewiring in the network) is changing in the most substantial way between the sub-populations analyzed. We note that in the datasets we studied, the genes that undergo the largest rewiring are not the central genes (genes with the highest degree of connectivity in the regulatory network). Central genes were studied in yeast and E. coli regulatory networks and, as expected, many of them were found to be essential, as shown in a number of knockout experiments [60]. However, we found that the combined expression profile of the top central genes of either of the sub-population networks is not a good proxy for the transcriptome-wide gene profile. Specifically, we compared the performance of a classifier based on the expression data of the ten top central genes with a classifier derived by utilizing the genome-wide expression profiles (data not shown). In most datasets, the classification error obtained using the classifier based on the top central genes is substantially higher. This might be attributed to the fact that samples of very similar cells from the sub-populations have similar regulatory networks. This implies that their central genes do not change their expression levels noticeably.

Our results suggest that genes whose collective expression profiles best differentiate between relevant disease conditions in cancer tend to localize on the transcriptional regulatory network. In other words, many of these differentially expressed genes have short gene-gene distances that can be depicted by relationships such as a "sibling relationship" (genes directly regulated by a common TF), a "cousin relationship" (genes regulated by different TFs that are regulated by a common TF), or genes that are closely localized in an "uncle-nephew relationship". Identification of these types of "families" or sub-networks gives us insight into the regulatory control that differentiates between normal and cancer cells or between good and poor prognosis patients. For instance, Figure 8 suggests an "uncle-nephew relationship" between Erbb2 and Bcl-2 via Myc or E2F1 through CUTL1. In addition, we can identify key regulators that impact the expression of large sets of genes implicated in cancer. For instance the CS networks for ALL patients reveal the close regulatory relationship of retinoic acid receptor with at least 7 of the genes that collectively separate ALL from AML (Figure 3).

The classification schemes introduced here utilize the topological properties of the network and facilitate the identification of key transcription factors that may be involved in gene dysregulation. Typically, these TFs do not appear in the short list of differentially expressed genes obtained in standard microarray analyses, but they are still linked to many of the genes in the short list. Therefore, these TFs may be good candidates for future *proteomic *biomarker screening tests, because a relatively small number of these TF markers (that have transcriptional linkage with the differentially expressed genes) may be effective for differentiating the samples due to differences in their protein expression levels, localization or phosphorylation status.

Although specific predictions about transcription factor-gene links can only be fully confirmed by further experimental studies, the success of using the networks to classify phenotypes using a wide range of cutoff parameters for binding and co-expression suggests that substantial parts of the putative networks we derived contain biologically relevant information. As a next step we plan to derive a more accurate TF-target gene binding connectivity network by integration of future ChlP-chip data and complex protein-DNA prediction algorithms (e.g. schemes that find motifs enriched in many promoters of genes that share common expression profiles across multiple experimental conditions, and take into account the conservation of these motifs in closely related organisms).

## Conclusion

In this work we introduced a novel approach of separating cell types by analyzing changes in the functionality of TF-target gene pairs (regulatory links) rather than changes in expression levels of individual genes (nodes). Moreover, we showed that other topological characteristics of the CS regulatory networks allow us to effectively classify cell types and patient samples. This approach enables us to identify key transcriptional circuitry alterations by finding pairs of regulating-regulated genes, whose coordinated expression activities undergo the most substantial modification from one class of patients to another. Inspection of the regulatory networks we constructed for cancer cells shows that genes that differentiate between states tend to localize on these networks. Despite the limitations of the currently available data from human DNA localization studies compared to that for model organisms, the network drafts we derived by intersecting expression data with a mix of predicted and experimental binding input already enable us to find key regulators and foci of deregulation within the cancer regulatory network.

## Methods

### Microarray datasets

1) Distinct normal cell types: Affymetrix U133 chip mRNA expression data of 10 resting neutrophil and 19 resting monocyte samples obtained from normal individuals [36].

2) Hematologic Malignancies: Affymetrix data comparing 47 samples obtained from acute lymphoblastic leukemia (ALL) patients to 25 samples from acute myeloid leukemia (AML) [23]. Affymetrix U133A chip mRNA expression data from 22 patients with myeloid leukemia involving a t(8;21) translocation with the AML/ETO fusion protein and 19 patients with myeloid leukemia involving a t(15;17) translocation with the RAR/PML fusion protein [43].

3) Diseased versus normal tissues: Affymetrix U133A and U133B chips of 9 renal cell carcinoma samples and matched normal samples [37].

4) Breast Cancer: Hu25K oligonucleotide arrays from 295 breast cancer patients (175 disease free and 120 with disease recurrence) [40,44,55], U95Av2 expression data from 22 breast cancer survivors and from 29 patients with recurrence [38], and U133A Affymetrix datasets from 183 patients who were disease free at 5 years versus 93 patients with recurrent disease [41].

### Simultaneous array and gene normalization

We applied a bi-normalization procedure to the expression data for gene profiling analysis, as described in previous work [36,61].

### Connectivity networks

The human and mouse connectivity networks were derived from a combination of high quality literature references (documented in the professional version of the Transcription Factors Database, TRANSFAC) and predictions based on matching known and putative transcription factors consensus binding sites sequences with the 1 kb (or 5 kb) upstream promoter regions of all human and mouse genes stored in the Goldenpath Database at UCSC [62]. We used the default parameters of the MATCH algorithm (provided by TRANSFAC) and a minimal score of 0.85 as a threshold for defining direct regulation connectivity between a transcription factor (TF) and a gene. The information is stored in a rectangular adjacency matrix, in which regulating TFs are represented by column indices and regulated genes by row indices. The elements of the matrix *C*_ij _are assigned a value of one if transcription factor *j *directly regulates gene *i*. If not, the elements are assigned a value of zero. We arranged the rows of this matrix such that the regulated genes in the first rows are TF genes. Furthermore, the order of the TFs across these rows is equivalent to their order across the columns. Thus, the upper square block of the matrix *C *consists of the TF regulatory network, and its non-zero diagonal terms *C*_ii _correspond to self-regulation. The lower rectangular block of this matrix represents the regulatory relationships between TFs and non-regulating genes.

### Integration of the connectivity network with co-expression networks

Essentially, to obtain condition specific networks we intersect two types of information: a) TF-target gene binding b) TF-target gene co-expression. A nonzero entry in the binding matrix represents predicted or known regulation of a gene by a given TF based on either: i) the degree of matching between the TF PWM with the promoter region of a potential target gene is greater than a predetermined threshold (predictive), ii) prior experimental knowledge of the regulation of the target gene by the TF as documented in TRANSFAC (please note that in the current version of TRANSFAC some of this knowledge includes several ChlP-on-chip experiments), or iii) ChlP-on-chip experimental data that are still not incorporated in TRANSFAC (as we implemented in the analysis shown in Fig. 7)

### Classification with feature selection

We perform feature selection to rank the links, genes and degree of the nodes that undergo the most substantial change between two classes (two cell types, normal versus cancer, survival versus recurrence). To rank the differential activity of the links we first concatenated the adjacency matrix representing the network of each sample to a one-dimensional vector, whose entries are assigned a value of 1,0, or -1. These values correspond to a stimulatory relationship, no relationship or inhibitory relationship of the TF-gene links, respectively. We then formed a matrix consisting of all of these vectors such that each column of this matrix represents the sample link vector. The number of rows (features) in this matrix is equal to the number of genes times the number of TFs involved in the corresponding two class microarray study. We then subjected this matrix to a meta-classifier that in the first step ranks the features according the information gain of using this feature to explain the class data in the training set. The features with the highest rank are then passed to train the base classifier (decision tree, Naïve Bayes, nearest neighbors etc.) using the training data only. To estimate the cross validation error rate of the classifier in the reduced feature space, we performed ten-fold cross validation ten times, using ten different seeds. We note that the feature selection is done on the training data only and not using the whole data, since the latter can lead to an optimistic bias in the accuracy estimates. To perform expression based classification and identify differentially expressed genes, we used an equivalent procedure to classify the samples by first reducing the dimensionality of the data using feature selection followed by classifier training and cross validation. Similarly, we formed a genome-wide profile indicating the in-degree (centrality) for all genes (or out degree of all TFs) in each individual network and searched for the subset of genes or TFs whose centrality measure is altered significantly between the two classes. We implemented the same procedure for these three types of input data (link profiles, gene profiles and gene centrality profiles) employing the WEKA package [63] via Matlab™ and using the following learning configuration: a) Information Gain for an attribute evaluator method, b) Ranker for a search method of attribute selection and c) nearest neighbor learner for a classifier. We classified these three types of data using many other combinations of feature selection and classifiers implemented in WEKA. The cross-validated classification error rates obtained by using the specific combination of feature selection and learning method reported in the results section were typically close to the performance obtained by the top classifiers tested for each of these datasets. Our goal here was to identify the prominent features that separate the data in an efficient manner. The rationale for the choosing Information Gain and Ranker was the large number of variables (two orders of magnitudes larger than the number of genes in the genome). This allows us fast computation of the merit of each feature. The following example shows how to evaluate the information gain of a feature that has three discrete states (+1,0,-1):

a) calculate the information measure for all the samples N = N_a _+ N_b _of class a and class b

info({N_a_, N_b_}) = -P_a _log2(P_a_) - P_b _log2(P_b_) where P_a _= N_a_/N and P_b _= N_b_/N

b) calculate the information measures in each of the discrete states (+1, 0, -1)

info({N^+1^_a_, N^+1^_b_}), info({N^0 ^_a_, N^0^_b_}), info({N^-1^_a_, N^-1^_b_}) where N_a _= N^+1^_a _+ N^0^_a _+ N^-1^_a _and N_b _= N^+1^_b _+ N^0^_b _+ N^-1^_b_

c) calculate the weighted average information value of these discrete states

info_w _= (N^+1^/N) info({N^+1^_a_, N^+1^_b_}) + (N^0^/N) info({N^0^_a_, N^0^_b_}) + (N^-1^/N) info({N^-1^_a_, N^-1^_b_]}, 

where N^+1 ^= N^+1^_a _+ N^+1^_b_, N^0 ^= N^0^_a _+ N^0^_b _and N^-1 ^= N^-1^_a _+ N^-1^_b_

d) the difference {info({N_a_, N_b_}) - info_w_} is the information gain of this feature.

The Ranker method ranks the attributes by their individual evaluations.

We calculate the information gain for each feature, and rank them according to this measure, which indicates the gain of information we obtain by classifying the data using this feature. Features above the 95^th ^percentile rank are kept unless their information gain is negligible with respect to the feature with the highest rank.

### Centrality

To examine the possibility of classifying the samples by network characteristics we used a feature space consisting of the degree of connectivity profile of all the nodes in the networks. Moreover, we questioned whether the collective expression of the central genes (hubs) in the regulatory networks is a good proxy for phenotype characterization. We tested four types of centrality measures: a) In-degree defined by the number of TFs that regulate a gene, b) Out-degree defined by the number of genes regulated by a TF c) In-PageRank (a measure invented by the Google Inc. founders) defined by the entries of the left eigenvector (with the largest eigenvalue) of the adjacency matrix representing the CS regulatory network. This measure of centrality factors in the extent that the regulated gene is connected to TFs that are centrally regulated as well, d) As in c but for regulating TFs using the right eigenvector of the adjacency matrix. We presented results using the In-degree centrality. Out-degree centrality produces somewhat higher error rates (data not shown). The PageRank measures resulted in very similar error rates (data not shown).

### Geodesic and gene-gene distances

The proximity of groups of genes is determined by the particular distance measures we used. The most straightforward distance measure we used on the directed graph representing the regulatory network is the geodesic distance. The geodesic distance between a gene-TF pair (gene_i_-TF_j_) is 1 if the corresponding entry in the adjacency matrix is one (*A*_ij _= 1). A pair of gene_i_-TF_j _whose *A*_ij _= 0 could be indirectly connected in the regulatory network via other transcription factors regulated by TF_j_. To find these indirect connections we reorganized the matrix *A*_ij _such that its upper square block, defined by *T*_ij_, consists of transcription factor pairs (TF_i_-TF_j_) only. If an entry of the adjacency matrix *A*_ij _is zero but the same entry of (*AT*)_ij _is nonzero, the geodesic distance between gene_i _and TF_j _is *2*. Similarly, if the entries of (*AT*^*m*^)_ij _for all *m *= 0,..,*n*-1 is zero and the corresponding entry (*AT*^*n*^)_ij _is nonzero the gene_i_-TF_j _geodesic distance is *n *+ 1. Thus, the geodesic distance between a TF and a gene is the shortest directed path between them, i.e., the smallest number of links connecting them. A pair of non-regulating genes has an infinite geodesic distance, because there is no directed path in the regulating network, which connects these genes. To define a distance between any pair of genes, whether any of these genes is a TF or not, we identify an "ancestor" transcription factor in the regulatory network, whose sum of geodesic distances to both genes is minimal. If one or two of the genes of a given pair is a TF, the gene-gene distance of this pair is defined by the either the shortest directed path between them or the gene-gene definition above, whichever is smaller.

## Authors' contributions

All authors participated in the design of the study and writing of the manuscript. DT and YK performed all the computational aspects of the work. All authors read and approved the final manuscript.

## Supplementary Material

Additional file 1 overview of the flow of network construction Click here for file

## Figures and Tables

**Table 1:** Classification error based on Network Links, Transcription Profile and Degree Error Estimates (ten-fold cross validation ten times, using ten different seeds)

Dataset	Network Link	Gene Expression	(In) Degree
**Leukemia**			

ALL/AML[23]	9%	4%	4%
Myeloid Leukemia [43]	6%	0%	0%

**Breast Cancer**			

Duke [38]	23%	17%	25.5%
Dutch [39, 44, 55]	43%	42%	39.8%

**Paired Cell Types**			

Renal Carcinoma vs. Normal Kidney [37]	0%	0%	0%
Monocytes vs. Granulocytes [36]	0%	0%	0%

**Table 2:** Examples of top network links or in degree of genes

**Dataset**	**Network Links**	**Genes with Differential In Degrees**
**Leukemia**		

ALL/AML	RUNX1→RAG1 CBFB→RAG1 RARA→ELA2 RARA→SERPING1 RARA→PFC RARA→MSH2 RARA→MPO	MGST1 HOXA9 DF PYGL CD33 CTSC PFC PTX3 VPREB1 POU2AF1 MLC1SA RAG1 CSTA LAMP2 MPO BLK ELA2 UPP1 MAN2B1 CXCL2 ACTA2 ANXA1 CD24 ZYX BAT1 PLEK GATA3

Myeloid Leukemia	NFKB1→F3 EGR1→F3 CEBPA→PPARG FOXO3A→RUNX1T1 MYC→LTC4S MAX→LTC4S MYC→HLA-DM	CDH7 NFKB1 ZNF516 BNIP2 STAT3 PLEKHM1 CPS1 CDC2 CD2 MT1DP HNF4A BLOC1S1 CRISP2 ARR3 EZH1 IGHGP EZH2

**Breast Cancer**		

Duke	NFKB1→BTRC FOXO3A→PLS1 BHLHB2→HGFBP6 POU2F1→TRAF3IP1 FOXJ2→MEP1A MEF2D→MEP1A	DRD2 RPA3 NDUFA6 NRF1 HBB CLCNKB PDE3A FKBP4 MFAP1 SURF1 PIM1 SLIT1 TAF1 DUSP4 CRYBB2 TNP2 MAPK7 LTBP1 TCEB2 SOX15 HIP2

Dutch	PAX8→FEN1 PAX8→E2F1 TCF2→KLHL5 FOXM1→TAD3 TCF2→ZBTB4 ELK1→UBE2C TCF2→UBE2C E2F1→CELSR2 PAX8→TK1 FOXM1→DPF2 FOXM1→CD160 FOXM1→CCL3 E2F1→UBE2Q USF1→MYBL2 FOXM1→ABCB1 E2F1→TK2 PBX1→TROAP	PSMB10 TCP10 BAK1 DPP6 PDGFRL ADCY7 SEC13L1 ALDH3A2 RAB5A HDAC3 PCTK1 PGC IFNA7 IHH ABCD3 PFKFB3

**Paired Cell Types**		

Renal Carcinoma vs. Normal Kidney	BHLHB3→IL15RA FOXO3A→PLK2 BHLHB3→HLX1 NR1H3→CDCA7L PAX2→KCNK10	ABCA4 AP2A2 ALDH3B2 AGXT AGRP ADCY2 ACOX1 ACP2 ABCA1 ABAT NAT1 ADCY8 AHSG ADCY3 PARP4 ACVR2 ADRA2A ACY1 AIM1

Monocytes vs. Granulocytes	PAX8→MARCO NR1H2→MARCO PAX8→MARCO CEBPA→MARCO CEBPG→MARCO RXRB→MARCO RARA→MARCO HIF1A→PRSS15 EGR2→RIS1 RARA→SLC30A6	MRPS16 CYP4F12 ARL4A GSS TPST1 PMPCA CTAGE5 HEY1 KIF20A PKN2 SLC30A6 DNAJD1 PRSS15 HBEGF MARCO NDUFA7

**Table 3:** Accuracy estimates (100% – error rate) using different base classifiers and feature selection techniques, based on twenty repetitions, each utilizing ten-fold cross validation for a total of 200 runs

**Base Classifler**			**IB1**		**NaiveBayes**		**KStar**		**J48**		**IBk**	
**Expression**	**Lower**	**Upper**	**Accuracy**	**SD**	**Accuracy**	**SD**	**Accuracy**	**SD**	**Accuracy**	**SD**	**Accuracy**	**SD**

Threshold	0.1	0.9	79.58%	4.79%	79.38%	3.86%	84.17%	2.72%	85.69%	1.63%	84.09%	2.64%
Threshold	0.2	0.8	91.56%	2.35%	90.47%	2.85%	91.67%	2.53%	88.50%	2.70%	93.75%	1.46%
Threshold	0.33	0.66	91.89%	2.95%	91.17%	2.39%	90.44%	2.90%	89.10%	2.88%	92.81%	1.91%
Absolute	50	200	90.28%	1.84%	90.74%	1.55%	90.46%	1.92%	88.54%	2.20%	90.62%	1.92%
Tanh	0.25	0.75	92.71%	2.43%	93.45%	1.92%	91.67%	2.13%	89.93%	3.46%	91.56%	1.19%

												

**Feature Selection**			**InformationGain**		**ChiSquared**		**GainRatio**		**Wrapper Subset**			

**Expression**	**Lower**	**Upper**	**Accuracy**	**SD**	**Accuracy**	**SD**	**Accuracy**	**SD**	**Accuracy**	**SD**		

Threshold	0.2	0.8	91.56%	2.35%	89.96%	2.74%	92.41%	1.72%	87.30%	3.36%		
Tanh	0.25	0.75	92.71%	2.43%	92.55%	2.72%	92.56%	2.34%				

Table 3 demonstrates explorations of the effects of varying the parameters involved in classification based on network features. Each pair of cells provides the overall accuracy and standard deviation (SD) for a parameter set based on twenty repetitions, each utilizing ten fold cross-validation, for a total of 200 runs. Table 3 displays the effects of variation of the base classifier combined with variations in the thresholds used to determine the condition specific expression state of genes. All classification was done using the WEKA package. Complete documentation of each method is available at the WEKA website [63]. Briefly, IB1 and IBk are nearest neighbor classifiers using 1 and k neighbors, respectively. Results here are reported for IBk with k = 3. J48 is a standard C4.5 decision tree algorithm implementation, and KStar is an instance-based classifier that differs from the nearest neighbor learners through its use of an entropy-based distance function. Parameters used to determine up- or down-regulation in the co-expression networks were at the 80^th ^and 20^th ^percentile of expression levels respectively or at absolute expression intensities of 200 and 50 (for Affymetrix Arrays only). Continuous co-expression matrix (labeled as Tanh in the table) was constructed by preprocessing the gene expression data *g *by the hyperbolic tangent transformation: G = tanh[(g-μ)/δ], where (μ and δ are the average and inter-quartile range of the expression level of all genes across all experiments respectively. The lower block in Table 3 shows the effects of different feature selection methods. The first three methods evaluate and rank the attributes. Results using information gain are reported in the main results of the text, and this method is described in the methods section. The chi-squared method calculated a chi-squared statistic with respect to the class; the gain ratio method evaluates attributes by calculating the ratio of the entropy of the class minus the entropy of the class conditional on the attribute to the entropy of the attribute. Additionally, a wrapper method was assessed in which feature subsets were explored utilizing a greedy forward hill-climbing method to search through the space of attribute subsets. In testing various methods of feature selection for the ALL vs. AML dataset, we find that the top ten links were identical for all methods tested, and over 90% of the top 25 links were selected by all methods tested.

**Table 4:** Accuracy estimates (100% – error rate) using different parameters for TFBS Identification, based on twenty repetitions, each utilizing ten-fold cross validation for a total of 200 runs

**Promoter Range**					**1 kb upstream**		**1 kb upstream**		**5 kb upstream**		**5 kb upstream**	
**PWM**					**All Proflies**		**Limited Profiles**		**All Proflies**		**Limited Profiles**	

**Classifier**	**Expression**	**Lower**	**Upper**	**Feature Selection**	**Accuracy**	**SD**	**Accuracy**	**SD**	**Accuracy**	**SD**	**Accuracy**	**SD**

IB1	Threshold	0.2	0.8	InfoGain	91.56%	235%	81.65%	4.22%	93.06%	1.80%	93.27%	2.35%
IB1	Threshold	0.33	0.66	InfoGain	91.89%	2.95%	90.72%	2.90%	95.57%	2.04%	93.62%	1.78%
IB1	Threshold	0.2	0.8	ChiSquared	89.96%	2.74%	81.00%	4.04%	93.92%	1.75%	92.63%	2.22%
IB1	Threshold	0.33	0.66	ChiSquared	91.10%	2.90%	90.67%	2.79%	94.07%	2.43%	93.43%	2.31%
IB1	Tanh	0.25	0.75	InfoGain	92.71%	2.43%	92.74%	2.30%	92.13%	2.47%	92.00%	3.01%
Naive Bayes	Threshold	0.2	0.8	InfoGain	90.47%	2.85%	8235%	3.78%	96.04%	1.34%	94.98%	1.41%
Naive Bayes	Threshold	0.2	0.8	InfoGain	91.67%	2.53%	83.18%	3.11%	94.39%	1.73%	93.78%	2.00%

Table 4 shows the effects of variations in the parameters for connectivity network construction. The genomic region searched for transcription factor binding sites was either 1000 bp or 5000 bp upstream of known genes. Two different collections of Position weighted matrices (PWM) were also applied: 1) all the matrices provided by TRANSFAC relevant to mammalian genes (All Profiles), or 2) the selection of PWMs identified by TRANSFAC as 'high quality' (Limited Profiles).
