# Supplementary figures and images for: Characterizing disease states from topological properties of transcriptional regulatory networks
Source: BMC Bioinformatics. 2006 May 2;7:236. doi: 10.1186/1471-2105-7-236 (PMC1482723; doi:10.1186/1471-2105-7-236)

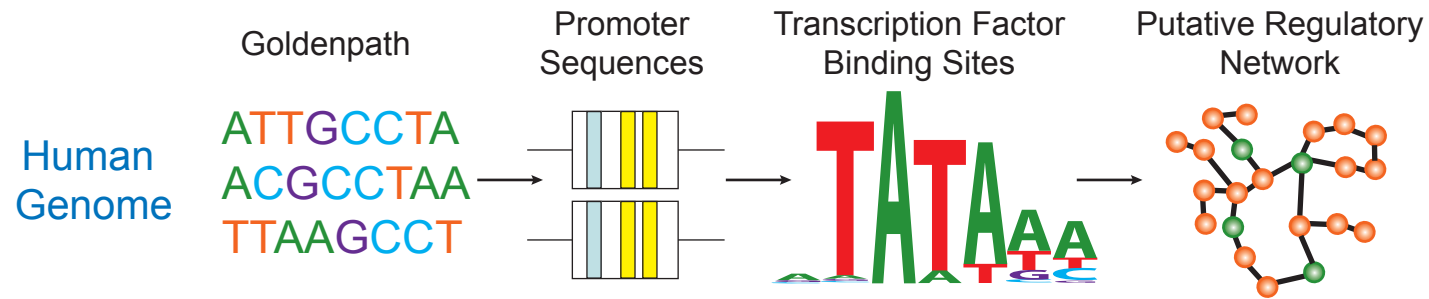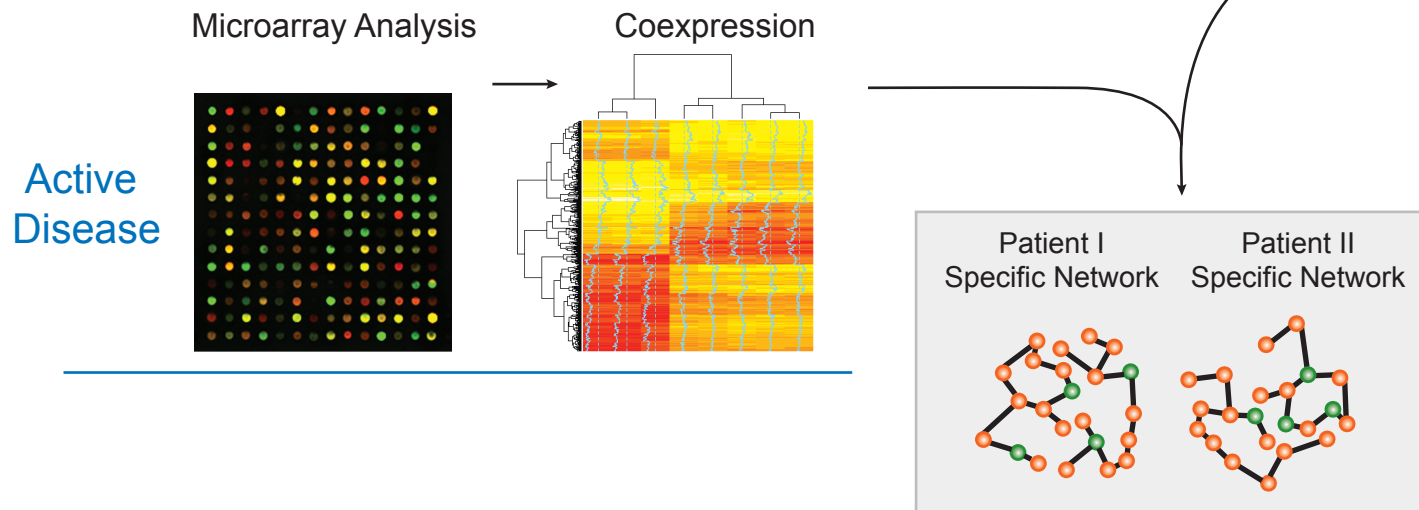

Supplement: Additional file 1 — overview of the flow of network construction [file 1471-2105-7-236-S1.pdf]
